# High-throughput analysis of leaf physiological and chemical traits with VIS–NIR–SWIR spectroscopy: a case study with a maize diversity panel

**DOI:** 10.1186/s13007-019-0450-8

**Published:** 2019-06-26

**Authors:** Yufeng Ge, Abbas Atefi, Huichun Zhang, Chenyong Miao, Raghuprakash Kastoori Ramamurthy, Brandi Sigmon, Jinliang Yang, James C. Schnable

**Affiliations:** 1 Department of Biological Systems Engineering, L.W. Chase Hall 203, University of Nebraska – Lincoln, Lincoln, NE 68583 USA; 2 College of Mechanical and Electrical Engineering, Nanjing Forestry University, Nanjing, China; 3 Department of Agronomy and Horticulture, University of Nebraska-Lincoln, Lincoln, NE USA; 4 Department of Plant Pathology, University of Nebraska-Lincoln, Lincoln, NE USA

**Keywords:** Hyperspectral, Plant phenotyping, Partial least squares regression, Support vector regression, Machine learning, Vegetation indices, Macronutrients

## Abstract

**Background:**

Hyperspectral reflectance data in the visible, near infrared and shortwave infrared range (VIS–NIR–SWIR, 400–2500 nm) are commonly used to nondestructively measure plant leaf properties. We investigated the usefulness of VIS–NIR–SWIR as a high-throughput tool to measure six leaf properties of maize plants including chlorophyll content (CHL), leaf water content (LWC), specific leaf area (SLA), nitrogen (N), phosphorus (P), and potassium (K). This assessment was performed using the lines of the maize diversity panel. Data were collected from plants grown in greenhouse condition, as well as in the field under two nitrogen application regimes. Leaf-level hyperspectral data were collected with a VIS–NIR–SWIR spectroradiometer at tasseling. Two multivariate modeling approaches, partial least squares regression (PLSR) and support vector regression (SVR), were employed to estimate the leaf properties from hyperspectral data. Several common vegetation indices (VIs: GNDVI, RENDVI, and NDWI), which were calculated from hyperspectral data, were also assessed to estimate these leaf properties.

**Results:**

Some VIs were able to estimate CHL and N (R^2^ > 0.68), but failed to estimate the other four leaf properties. Models developed with PLSR and SVR exhibited comparable performance to each other, and provided improved accuracy relative to VI models. CHL were estimated most successfully, with R^2^ (coefficient of determination) > 0.94 and ratio of performance to deviation (RPD) > 4.0. N was also predicted satisfactorily (R^2^ > 0.85 and RPD > 2.6). LWC, SLA and K were predicted moderately well, with R^2^ ranging from 0.54 to 0.70 and RPD from 1.5 to 1.8. The lowest prediction accuracy was for P, with R^2^ < 0.5 and RPD < 1.4.

**Conclusion:**

This study showed that VIS–NIR–SWIR reflectance spectroscopy is a promising tool for low-cost, nondestructive, and high-throughput analysis of a number of leaf physiological and biochemical properties. Full-spectrum based modeling approaches (PLSR and SVR) led to more accurate prediction models compared to VI-based methods. We called for the construction of a leaf VIS–NIR–SWIR spectral library that would greatly benefit the plant phenotyping community for the research of plant leaf traits.

## Background

High-throughput plant phenotyping deals with rapid and large-scale collection of plant phenotypic data using advanced sensing, robotics and data analytics [1]. The ultimate motivation of research in this field is to collect plant phenotypes with the efficiency and resolution comparable to plant genomic data to facilitate gene discovery and targeted crop improvement [2, 3]. In the past few years, rapid advancements have been made in measuring morphological and structural traits of plants (height, size, leaf area, etc.) using imaging and image analysis [4, 5]. Time sequences of these nondestructive measurements further enable quantification of dynamic traits such as growth and stress response [6, 7]. To date, however, fewer studies have focused on high-throughput phenotyping of chemical compositions of plants. RGB (or visible light) cameras do not provide wide enough spectral ranges or high enough spectral resolution for chemical imaging, whereas hyperspectral imaging is still at its beginning stage for whole plant phenotyping, with a number of practical challenges yet to overcome [8].

Visible (VIS, 400–700 nm), near infrared (NIR, 700–1100 nm), and shortwave infrared (SWIR, 1100–2500 nm) spectroscopy (VIS–NIR–SWIR) is a promising technique to measure plant leaf physiological and chemical properties rapidly and non-destructively [9, 10]. In plant leaf cells, photosynthetic pigments such as chlorophylls and carotenoids absorb strongly in the VIS region. Water in fresh plant leaves interact with VIS–NIR–SWIR energy in two ways: strong reflection in NIR (due to the multiple reflections of turgid cell structure) and absorption in SWIR (in particular near 1450 and 1900 nm bands) [11]. Dry matter in plant leaves is composed primarily of organic compounds (structural carbohydrates, proteins, amino acids, etc.) that cause various spectral signals (combinational and overtone vibrational bands) in the SWIR region. In principle, these interactions between plant leaves and VIS–NIR–SWIR energy make it possible to measure chemical compositions of plant leaves nondestructively.

VIS–NIR–SWIR hyperspectral data has been widely used to calculate narrow-band vegetation indices (VIs, [12]). A VI is formulated from two or more spectral bands in a simple mathematical form. The selection of spectral bands is often based on the empirical relationship between given leaf properties and spectral data as determined by correlation analysis. Normalized difference vegetation index (NDVI) is one such index that is widely used for leaf chlorophyll and nitrogen analysis [13, 14]. The selection of VI bands can also be based on certain physiological aspect of plants. For example, Photochemical Reflectance Index (PRI) is devised to capture subtle spectral differences between the different carotenoid pigments involved in the xanthophyll cycle and can be employed as a proxy to estimate a plant’s radiation use efficiency [15]. One advantage of VI is that they are easy to compute. However, they discard a lot of spectral information which could otherwise be useful for modeling and prediction. With rapid advancements in computational capability in recent years, it is now practical to employ more advanced analytical tools to model the whole VIS–NIR–SWIR hyperspectral data for estimation.

A number of studies have explored the use of VIS–NIR–SWIR hyperspectral data in the context of plant phenotyping. Yendrek et al. [16] investigated the performance of VIS–NIR–SWIR to predict a number of photosynthetic and biochemical traits in maize plants grown under varying CO_2_ concentrations and varying nitrogen treatments. They found leaf chlorophyll and nitrogen contents could be best predicted, followed by specific leaf area, saturated rate of photosynthesis, maximum rate of phosphoenolpyruvate carboxylation, and leaf oxygen radical absorbance capacity. Heckmann et al. [17] evaluated the potential of leaf reflectance to measure three leaf properties of Brassica and maize plants including initialize slope of the A–Ci curve, maximal assimilation rate of CO_2_, and CN ratio. Silva-Perez et al. [18] measured 76 wheat genotypes grown in greenhouses and fields with a VIS–NIR–SWIR instrument and a gas exchange device. They reported model prediction (R^2^) of 0.62 for maximum Rubisco activity normalized to 25 °C, 0.7 for electron transport rate, 0.81 for SPAD, 0.89 for leaf dry mass per area, and 0.93 for nitrogen per unit leaf area.

In this paper, we evaluated the usefulness of VIS–NIR–SWIR hyperspectral data to estimate leaf physiological and chemical properties of maize plants from a maize diversity panel. The entire panel was grown three times, in the field under a nitrogen sufficient (+ N) and nitrogen deficient (− N) condition, and then in the greenhouse under an optimal condition. The leaf properties studied were chlorophyll content, water content, specific leaf area, and macronutrient concentrations of nitrogen, phosphorus, and potassium.

## Materials and methods

### Experiment and data collection

We used the maize diversity panel, which consists of 282 genetically diverse lines [19]. This panel was selected to capture as much of the genetic diversity present in maize as possible, while consisting of lines that could be reliably grown to maturity in temperate North America [19]. This panel has also been phenotyped for a wide range of traits across many years and environments.

The field experiment was conducted on Havelock Research Farm of the University of Nebraska-Lincoln (45°51′49″N, 96°31′09″W). The predominant soil types were Zook silty clay loam and Colo silty clay loam. The maize diversity panel was grown in two replicates, one under low nitrogen condition (− N) and the other normal condition (+ N). For the + N treatment, 135 kg/ha urea (dry fertilizer) was applied; whereas for the − N treatment, no N fertilizer was added. The planting date was May/16/2018. Each replicate consisted of 288 plots, among which 229 were from the maize diversity panel. The remaining plots were hybrid check varieties (B73xMo17 and B37xMo17) and expired plant variety protection (PVP) lines interspersed randomly. Each plot was 1.6 m wide and 6.3 m long, comprising of two rows of 38 seeds from each maize line. All other agronomic practices followed the recommendations by University of Nebraska’s Research Farm support group.

Plant leaf sampling was conducted on July/12 and July/13 2018, when roughly 50% of the plots were tasseling or had already tasseled. From each maize genotype (plot), a representative plant was identified. Leaf 2, 3 and 4 (leaf 1 was the flag leaf) from the plant were cut at the stem and immediately placed in a Ziploc bag and stored in an ice cooler. The leaf samples were then transported to the lab and processed and analyzed for leaf chemical properties.

VIS–NIR–SWIR reflectance spectra of leaf samples were measured by a benchtop spectroradiometer (FieldSpec4, Malvern Panalytical Ltd., Formerly Analytical Spectral Devices) with a contact probe. The spectral range of the instrument was 350–2500 nm and the spectral sampling interval was 1 nm. Each raw spectrum therefore had 2151 data points. The contact probe had a light aperture of 10 mm, which was its effective sampling area. For each leaf, three spectral measurements were taken at the tip, middle and base sections (but avoiding the midrib area) to account for in-leaf variability. Measurements were also made consistently from leaf’s adaxial side. The nine VIS–NIR–SWIR scans were then averaged to represent the spectral reading from that plant.

Leaf chlorophyll concentration (CHL) was measured with a handheld chlorophyll concentration meter (MC-100, Apogee Instruments, Inc., Logan, UT) using the sensor’s build-in calibration for maize. Similar to the VIS–NIR–SWIR measurements, chlorophyll concentration was also measured at three locations per leaf and nine readings from each plant were averaged. The unit of CHL was µmol/m^2^.

Leaf area (LA) was measured with a leaf area meter (LI-3100, LI-COR Biosciences, Lincoln, NE). Fresh weight (FW) of the leaves was recorded by a digital balance. Leaf samples were placed in a walk-in oven set to 50 °C and dried over 72 h to a constant weight. Dry weight (DW) of the leaves was then recorded. Leaf Water Content (LWC, %) was calculated as (FW–DW)/FW × 100%. Specific Leaf Area (SLA, m^2^/kg) was calculated as LA/DW.

Dried plant samples were sent to a commercial lab (Midwest Laboratories, Inc., Omaha, NE) where the samples are ground, homogenized, and analyzed for N, phosphorus (P), and potassium (K) concentration. N was analyzed with Dumas method using a LECO FP428 nitrogen analyzer (AOAC method 968.06). P and K were analyzed with microwave nitric acid digestion followed by inductively coupled plasma spectrometry (AOAC method 985.01).

A third replicate of the maize diversity panel was grown at the University of Nebraska-Lincoln’s Greenhouse Innovation Center. Three seeds were sown in 9.08 L pots (diameter 24 cm, height 26 cm) and thinned to one plant per pot after germination. Temperature in the greenhouse was set between 22.7 and 28.3 °C; and relative humidity was approximately 60%. The lighting cycle was set at 16 h from 0600 to 2200 hours. The pot was filled with growth media (Premier Tech Horticulture Promix BX) mixed with 0.015 kg of 15-9-12 osmocote (3–4 months release), 0.015 kg of 15-9-12 osmocote (5–6 months release), 0.037 kg of lime, and 1.3 kg of water. Water was added daily to pots with automated watering stations, with a target weight of 7.4 kg (including the pot carrier) at the beginning and 8.3 kg at the end. The date of planting was Aug/1/2018 and the leaf samples were taken on Oct/9/2018 and Oct/10/2018 (plants were at the flowering stage) following the same protocols as the field samples described above. The total number of samples from the greenhouse was 262, which included 229 lines from the maize diversity panel and 33 maize landraces.

In summary, the six leaf physiological and chemical properties we were interested in VIS–NIR–SWIR modeling were: leaf chlorophyll concentration (CHL, µmol/m^2^ of leaf area), leaf water content (LWC, %), specific leaf area (SLA, m^2^/kg), nitrogen (N, %), phosphorus (P, %) and potassium (K, %). CHL, LWC, SLA and N were among the most important leaf properties frequently studied by plant breeders, physiologists, and agronomists. While P and K were less studied spectroscopically, both were essential nutrients that have significant implications for crop production.

### Spectral preprocessing and multivariate modeling

Spectral data from 350 to 450 nm exhibited relatively high levels of noise, and were removed and excluded from downstream spectral analysis. The spectra were preprocessed with a Savitzky–Golay smooth filter to further reduce noise (window size = 5 and polynomial order = 2, [20]. The smoothed spectra were down-sampled to every five nm to reduce the dimensionality of the predicator variables for more efficient computation.

The entire sample set was randomly split into a training set (60%) and a test set (40%). The training set was used for calibrating prediction models of the six maize leaf properties using spectral data; and the model performance was assessed on the test set. We investigated two multivariate modeling approaches: Partial Least Squares Regression (PLSR) and Support Vector Regression (SVR). Both approaches were widely employed for modeling by using all wavebands in VIS–NIR–SWIR hyperspectral data. PLSR is a linear modeling technique where the regression is conducted between the response variable and the PLS Latent Variables (LV). The LVs are linear combinations of the original wavebands which achieve (1) accounting for the maximum variability in the hyperspectral data, and (2) maximally correlated with the response variable (Helland [21]). SVR, on the other hand, is a nonlinear technique where an optimal hyperplane is constructed in a higher dimensional feature space. A linear regression function is then computed in the higher dimensional feature space for the original wavebands which are mapped through a kernel function [22, 23]. PLSR and SVR, together with other techniques (such as Random Forest and Artificial Neural Network) are usually referred to as Machine Learning approaches [24].

Before modeling, response and predictor variables were zero-centered (by their respective means) and scaled to unit variance (by their respective standard deviations). Ten-fold (random segments) cross validation was employed in model training to balance model complexity and predictive accuracy (i.e., avoid overfitting). In PLSR, models having as many as 25 latent variables (n_LV_) were considered, and the best model was the one that gave the lowest cross-validated root mean squared error (RMSE_CV_). In SVR, a linear kernel function was used. The regularization parameter C (cost for constraints violation) was tested with five values: 0.01, 0.1, 1, 10, and 100; and the optimal C was the one that gave the lowest RMSE_CV_ in cross validation.

The best models were then applied to the test set. The models were evaluated by comparing the lab-measured and model-estimated leaf properties using Coefficient of Determination (R^2^), Root Mean Squared Error of Testing (RMSE_T_, Eq. 1), Mean Absolute Percent Error of Testing (MAPE_T_, Eq. 2) and Ratio of Performance to Deviation (RPD, Eq. 3). These analyses were performed in R statistical environment [25] with the “pls” [26], “prospectr” (Stevens and Ramirez-Lopez [27]), and “e1071” [28] packages

1 $$RMSE = \sqrt {\frac{1}{N} \times \mathop \sum \limits_{i = 1}^{N} \left( {\hat{Y}_{i} - Y_{i} } \right)^{2} }$$

2 $$MAPE = \frac{{\frac{1}{N} \times \mathop \sum \nolimits_{i = 1}^{N} \left| {\hat{Y}_{i} - Y_{i} } \right|}}{{\bar{Y}}}$$

3 $$RPD = \frac{SD}{RMSE}.$$

### Vegetation indices

Hyperspectral-based, narrow-band VIs are commonly used to quantify leaf CHL, N and LWC. To test the usefulness of the VIs for predicting the leaf properties in our dataset, we computed three common VIs from the VIS–NIR–SWIR hyperspectral data. They were Green Normalized Difference Vegetation Index (GNDVI, [29], Red-edge Normalized Difference Vegetation Index (RENDVI, [30], and Normalized Difference Water Index (NDWI, [31]. GNDVI and RENDVI were shown useful for CHL and N quantification [32, 33], and NDWI useful for foliar water content [34].

Similar to the PLSR and SVR analyses, we used the training set (60%) to develop calibration models (linear regression considering a linear and quadratic term) between the leaf properties and the VIs, and then applied the models on the test set and reported test R^2^ and RPD. In addition, we also conducted an exhaustive search of all possible two-band combinations in the form $\left( {B1 - B2} \right)/\left( {B1 + B2} \right)$ (note GNDVI, RENDVI and NDWI all took this form to compute) and selected the one giving the highest correlation with the target leaf property to test its performance.

## Results

**Fig. 1:**
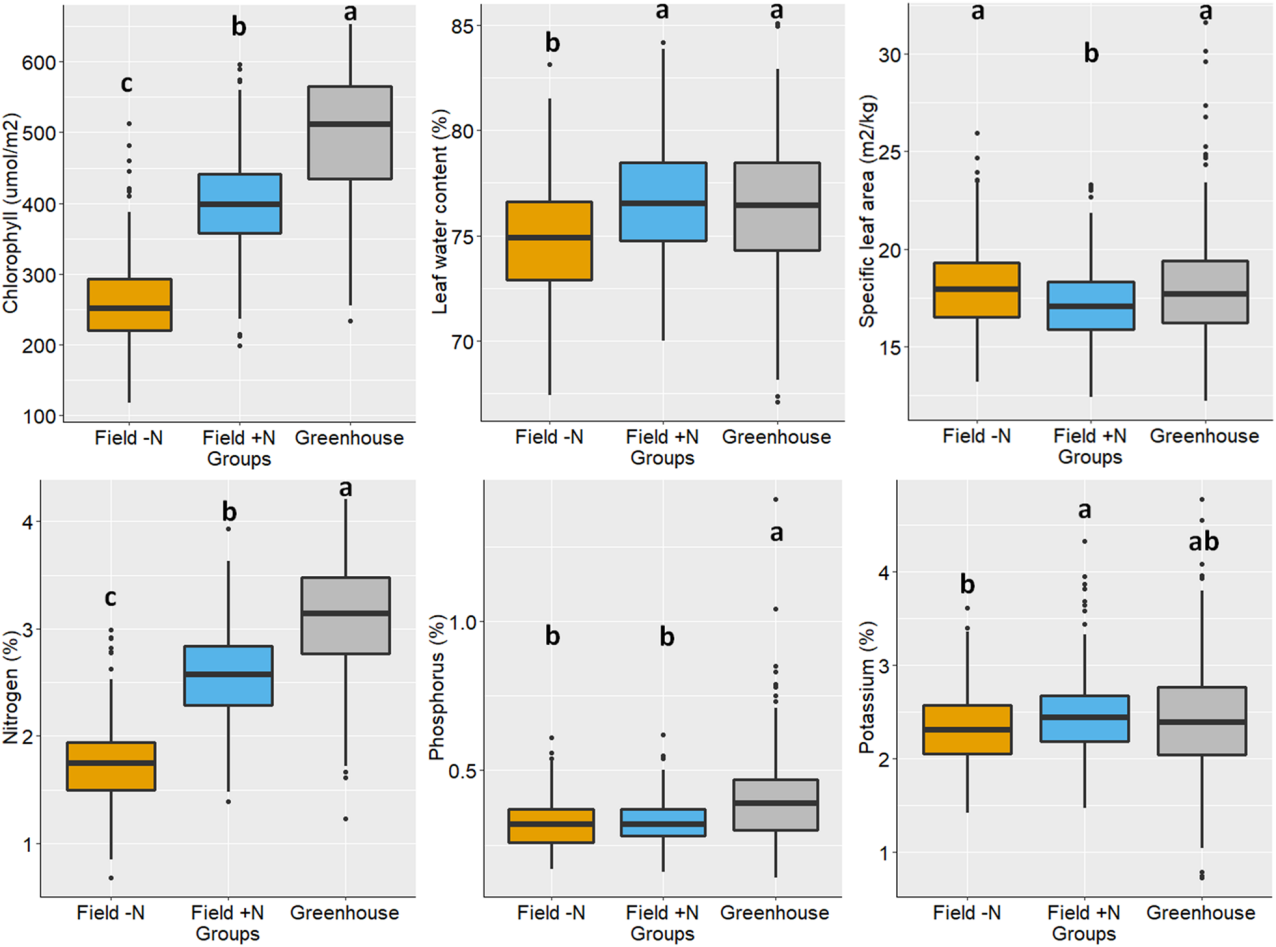
Boxplots comparing the leaf properties of maize plants from Field− N, Field + N, and greenhouse groups. The groups assigned to different letters indicated their means were different by Tukey’s Honest Significant Difference test (*p* value < 0.05)

Boxplots that compared the six leaf properties from the Field− N, Field + N, and Greenhouse groups are given in Fig. 1. It can be seen that there were significant differences in CHL and N among the three groups (Greenhouse being highest and Field− N lowest). This was expected because plants were continuously supplied with nitrogen throughout their lifecycle in the greenhouse; whereas N was limited in the field (in particular for the Field− N group). CHL was usually correlated with N and accounted for over 50% of N content in plants’ leaf tissue. For the other four leaf properties, the differences among the groups were smaller. The Field− N group tended to exhibit lower values for these four properties as well (except for SLA for which Field + N was lowest) although the difference was not always statistically significant.

**Fig. 2:**
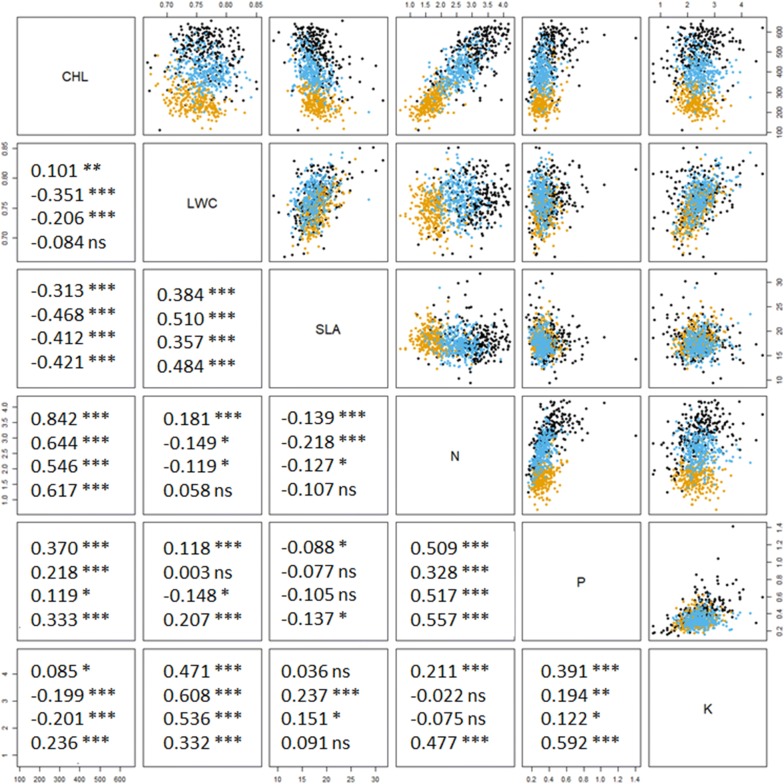
The matrix of scatterplots and Pearson’s correlation coefficients among the six maize leaf properties. The orange dots are plants in Field− N; blue dots are in Field + N; and black dots are in greenhouse. The correlation coefficients in the top row were calculated using the plants in Field− N, second row Field + N, third row greenhouse, fourth row by pooling the three groups together. Significance level: *** at 0.001 level, ** at 0.01 level, * at 0.05 level, ns not significant

Figure 2 gives the pairwise correlations among the six leaf properties. Strong and positive correlations were observed for CHL versus N, LWC versus SLA, LWC versus K, and N versus P; whereas strong and negative correlations were observed for CHL versus SLA. Note these correlations were consistent among the three different environments (Field− N, Field + N and Greenhouse), as well as when all the environments were considered together. On the other hand, other pairwise correlations were varying and inconsistent. For instance, significant negative correlation were observed between CHL and LWC for Field + N and Greenhouse. But when all data points were pooled together, there was not significant correlation between the two variables. This correlation structure among the six leaf properties revealed the complex interaction between genotypes and environments.

**Fig. 3:**
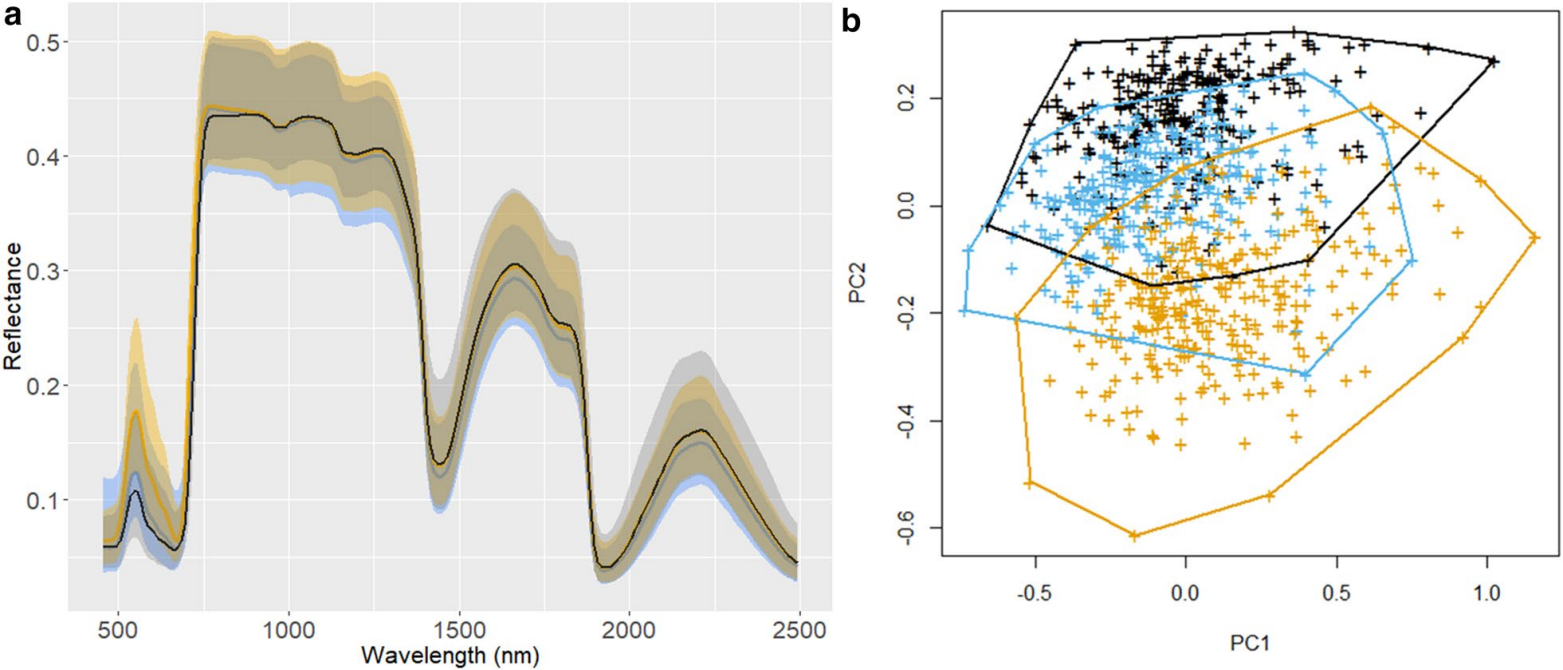
**a** The mean VIS–NIR–SWIR leaf spectra of the maize plants from Field− N (solid orange), Field + N (solid blue), and greenhouse (solid black). The bounding envelopes are the maximum and minimum spectra showing the spectral variability within each group. **b** Principal component score plots (PC1 vs. PC2) of each group and their convex hulls

Figure 3a shows the VIS–NIR–SWIR hyperspectral reflectance (after preprocessing) of maize leaves from the three groups. The reflectance spectra exhibited several typical features of fresh plant leaves [11, 35]: (1) low reflectance at blue and red bands due to strong absorption by the photosynthetic pigments, (2) the red edge, and (3) a series of local reflectance minima due to water absorption (at 970, 1240, 1450 and 1900 nm). The largest difference among the groups was in the VIS region (450–700 nm), where the Field− N group showed highest reflectance, followed by the Field + N group and then the Greenhouse group. This difference agreed with the significant difference in CHL among the three groups (Fig. 1). The difference of the mean spectra in the NIR–SWIR region, however, was quite subtle. We further conducted principal component (PC) analysis of hyperspectral reflectance data. The first PC score (accounted for 62.1% of the total variance in the hyperspectral data) versus second PC score (23.4%) is plotted in Fig. 3b along with the convex hull of each group. The spread within each convex hull could be regarded as the spectral variation caused by maize genotypes, whereas the distance between convex hulls could be regarded as the spectral variation attributable to the environments (i.e., Field− N, Field + N, and greenhouse). The partial overlaps among the three groups suggested that both genotype and environment contributed to the total variation in leaf VIS–NIR–SWIR hyperspectral data, and their contributions were confounded and likely not easily separable.

**Fig. 4:**
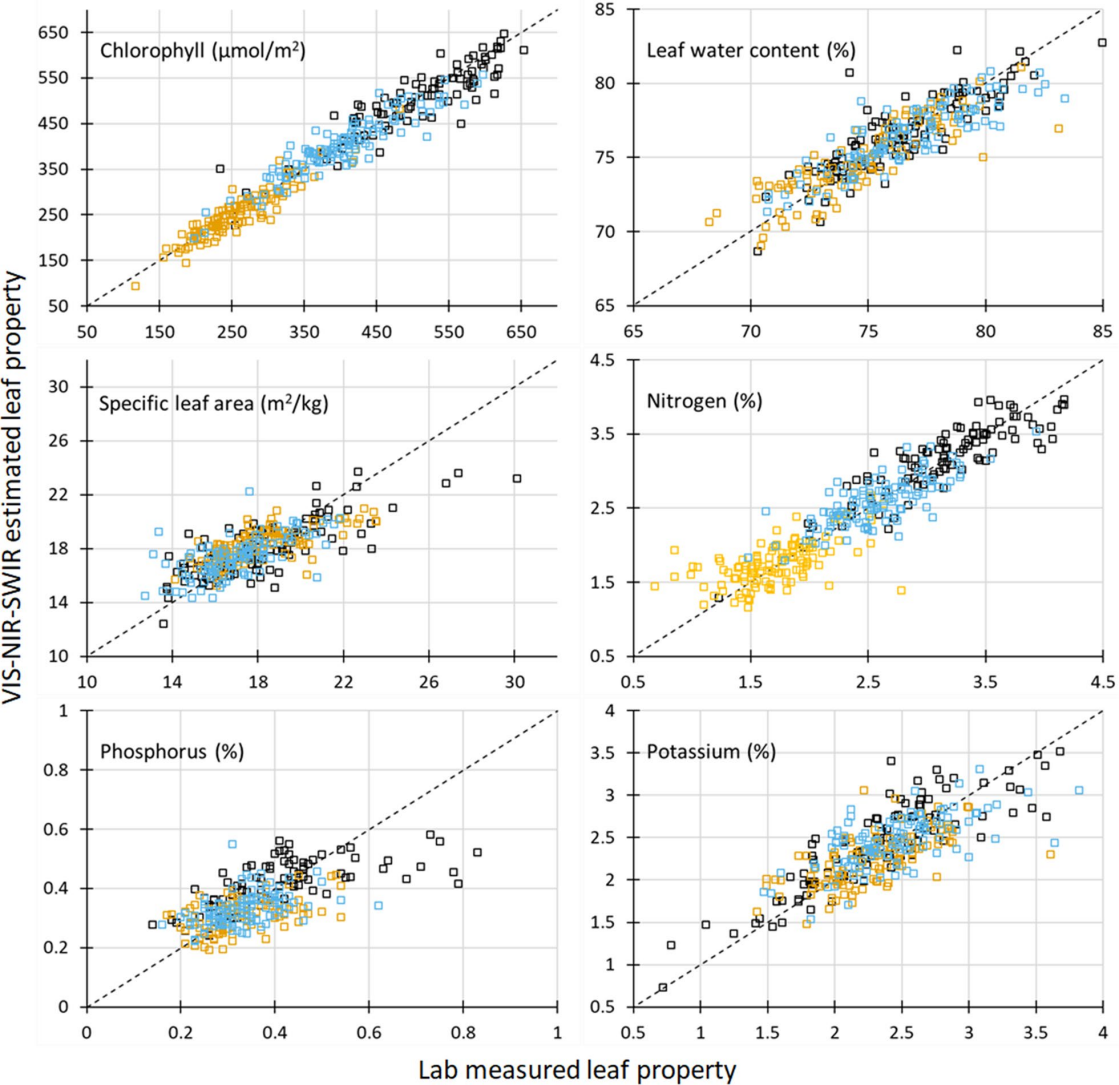
Lab-measured versus VIS–NIR–SWIR predicted maize leaf properties for the 40% test set. The orange squares are plants from Field− N; blue squares are plants from Field + N; black squares are plants from greenhouse. The black dashed line is 1:1 line. Statistics for the predictions can be found in Table 1

Table 1 summarizes the calibration and test results of estimating the six maize leaf properties from the VIS–NIR–SWIR hyperspectral reflectance data using PLSR. CHL was estimated by VIS–NIR–SWIR most successfully, with test R^2^ of 0.94 and model RPD of 4.12. N and LWC were also estimated quite satisfactorily, with test R^2^ of 0.86 and 0.70 and model RPD of 2.64 and 1.83, respectively. K and SLA were the next tier with moderate success. Their test R^2^ was greater than 0.5 and model RPD was close to 1.50. Finally, P was predicted with least success among the six leaf properties, with test R^2^ of 0.43 and model RPD of 1.33. Figure 4 shows the prediction scatterplots of the test set by the PLSR method, to provide visual indication of how good these predictions were.

**Table 1:** Calibration and test results of estimating leaf physiological and chemical properties of maize plants from VIS–NIR–SWIR hyperspectral reflectance spectra using Partial Least Squares Regression

Leaf properties	Calibration			Test			
	R^2^	RMSE_C_	n_LV_	R^2^	RMSE_T_	MAPE_T_ (%)	RPD
Chlorophyll (µmol/m^2^)	0.948	27.4	15	0.942	29.8	5.86	4.12
Leaf water content (%)	0.757	1.44	14	0.701	1.59	1.52	1.83
Specific leaf area (m^2^/kg)	0.578	1.55	12	0.554	1.61	6.80	1.50
Nitrogen (%)	0.869	0.252	18	0.855	0.282	8.82	2.63
Phosphorus (%)	0.453	0.084	18	0.435	0.084	16.8	1.33
Potassium (%)	0.705	0.272	25	0.586	0.301	9.76	1.54

The results of SVR modeling was similar to those of PLSR modeling (Table 2). Specifically, CHL was predicted best. N and LWC were predicted satisfactorily, followed by the moderate model performance with K and SLA. P still showed poor performance.

**Table 2:** Calibration and test results of estimating leaf physiological and chemical properties of maize plants from VIS–NIR–SWIR hyperspectral reflectance spectra using support vector regression

	Calibration			Test			
	R^2^	RMSE_C_	C	R^2^	RMSE_T_	MAPE_T_ (%)	RPD
Chlorophyll (µmol/m^2^)	0.950	27.0	1	0.946	28.5	5.59	4.30
Leaf water content (%)	0.765	1.42	1	0.703	1.81	1.58	1.83
Specific leaf area (m^2^/kg)	0.600	1.52	1	0.562	1.61	6.71	1.50
Nitrogen (%)	0.882	0.240	10	0.861	0.277	8.69	2.67
Phosphorus (%)	0.545	0.077	100	0.481	0.081	16.4	1.38
Potassium (%)	0.740	0.256	100	0.543	0.317	10.2	1.46

For all leaf properties, models performed only slightly better (R^2^ and RMSE) on the calibration set than the test set (Tables 1, 2). This suggested that the models were not overfitted to the training set, and they could be used with confidence to the new samples that are similar to the samples in this study.

In the study of field grown maize plants under N ample or limiting conditions, Yendrek et al. [16] achieved R^2^ of 0.85, 0.96, and 0.68 for CHL, N, and SLA prediction using VIS–NIR–SWIR hyperspectral data. In the study of wheat, Silva-Perez et al. [18] obtained validation R^2^ of 0.89 for leaf dry mass per area (a variable defined the same as SLA), 0.70 for N, and 0.65 for P. The results of these studies in general agreed with our results, showing very good to moderate prediction performance for leaf CHL, N, SLA, and P using VIS–NIR–SWIR hyperspectral data. Note that we did not find previous results on K and LWC prediction using VIS–NIR–SWIR with PLSR in the context of plant phenotyping.

RPD (Eq. 3) was a normalized index by considering the intrinsic variation of the dataset (standard deviation) and a model’s predictive accuracy (RMSE). It was often used to compare the models across different variables and studies. Following a few guidelines in the literature [36, 37], we proposed the following four RPD ranges to evaluate the performance of VIS–NIR–SWIR models for the phenotyping of plant leaf properties.

1. RPD > 3.5, Excellent. These models can be used for quantitative prediction with high confidence. If sufficiently tested, these models can potentially replace tedious lab-based analysis. Our CHL model fell into this category.
2. 2.5 < RPD < 3.5, Very Good. These models may also be used for quantitative analysis, but not with the level of confidence in the first category (certainly not replace lab-based analysis). Our N model was in this category.
3. 1.5 < RPD < 2.5, Good. These models are not for quantitative prediction, but can be used for qualitative screening (e.g., differentiate highs and lows from a large sample set, which are common for plant phenotyping and breeding). Our LWC, SLA and K models were in this category.
4. RPD < 1.5, Fair. These models may not be useful and should be further investigated and improved. Our P model was in the category.

Leaf properties like CHL and N could be estimated quite satisfactorily with GNDVI and RENDVI (Table 3, test R^2^ ranging from 0.68 to 0.85); whereas the estimation of LWC, SLA, P and K with these two VIs were poor (test R^2^ equal or lower than 0.1). This was in agreement with the literature where GNDVI and RENDVI were demonstrated to quantify plant CHL and N at both leaf and canopy scales; but their use for the other four leaf properties was not reported. Surprisingly, LWC could not be estimated successfully with any VIs, including NDWI. One possible reason was that the spread of LWC in our dataset was not large enough to build a robust model for NDWI. VIs computed from the best two band combination performed better for all leaf properties than GNDVI, RENDVI and NDWI. Slight improvement was achieved for CHL and N. LWC and SLA showed the largest improvement (test R^2^ of 0.43 and 0.31). The selected bands appeared in the longer wave SWIR region, indicating the usefulness of this spectral region in estimating LWC and SLA. Estimation of P and K was also slightly better for the best two band combination (R^2^ around 0.14), but still quite poor.

**Table 3:** Test results of using the selected vegetation indices (GNDVI, RENDVI, NDWI, and the best two band combinations) computed from VIS–NIR–SWIR hyperspectral data to predict the six maize leaf properties

Leaf properties	GNDVI (550 and 800 nm)		RENDVI (705 and 750 nm)		NDWI (860 and 1240 nm)		Best two band combination		
	R^2^	RPD	R^2^	RPD	R^2^	RPD	R^2^	RPD	Selected bands
Chlorophyll (µmol/m^2^)	0.847	2.56	0.805	2.27	0.063	1.03	0.921	3.54	730, 770 nm
Leaf water content (%)	0.045	1.02	0.045	1.02	0.094	1.05	0.428	1.32	1465, 2125 nm
Specific leaf area (m^2^/kg)	0.057	1.03	0.050	1.03	0.058	1.03	0.314	1.21	1870, 2275 nm
Nitrogen (%)	0.717	1.88	0.685	1.78	0.139	1.07	0.751	2.00	735, 745 nm
Phosphorus (%)	0.101	1.05	0.083	1.04	0.013	1.01	0.147	1.08	850, 860 nm
Potassium (%)	0.006	0.99	0.002	0.98	0.087	1.03	0.143	1.05	1215, 1325 nm

## Discussion

### Hyperspectral vegetation indices versus whole-spectrum based modeling (PLSR and SVR)

There was a clear advantage of using the whole-spectrum based approaches (PLSR and SVR) over VIs to predict leaf properties (Tables 1, 2 vs. Table 3). For CHL and N which VIs could predict satisfactorily, their models developed by PLSR and SVR performed even better. For the other four leaf properties which VIs predicted only fairly or poorly, PLSR and SVR still yielded moderately satisfactory predictions. As stated before, the advantage of hyperspectral, narrow-band VIs is computational simplicity. However, by selecting only a few (usually two) bands from hundreds or thousands of hyperspectral bands, a lot of useful information is discarded. The plant leaf is a complex mixture of many chemical compounds (such as water, pigments, N-containing proteins, structural carbohydrates, etc.), and they all contribute to the overall shape of leaf spectra. This is particular true when a diverse set of plants (like ours from a maize diversity panel) is studied. In addition, the physical state of the leaf (such as leaf thickness and surface roughness) also affect it reflectance spectra. Using PLSR and SVR that employ the entire spectra makes the models more flexible in accentuating the spectral features that are correlated with the target property while suppressing the bands whose variation is sensitive to other confounding factors. With the rapid advancement of computing, PLSR and SVR modeling can be done very efficiently. Moreover, other machine learning approaches such as Random Forest, Artificial Neural Network, and Ridge/Lasso regression can also be considered, giving researches a wide range of choices for their data. Some of these approaches might work particularly well under certain conditions. We therefore suggested that whole-spectrum based modeling should be used for the phenotyping of plant leaf physiological and biochemical traits using VIS–NIR–SWIR, as we virtually have no computational barriers that earlier researchers were facing.

### Advantages of VIS–NIR–SWIR hyperspectral data for plant phenotyping

Compared to high-throughput phenotyping of plant morphological traits using imaging techniques, phenotyping of plant leaf physiological and chemical traits lags behind. Destructive leaf tissue sampling followed by lab-based analyses remain as the mainstream method. VIS–NIR–SWIR has several advantages that make it powerful for phenotyping physiological and biochemical traits at the leaf level.

Firstly, it is rapid, nondestructive, and takes only a few seconds to acquire a scan. Rapidity enables fast screening of hundreds of plants or genetic lines, which is essential for high-throughput phenotyping. Non-destructiveness, on the other hand, allows repeated measurements of the same leaves and plants along their life cycle. These measurements would potentially lead to the quantification of more complex and dynamic traits such as nutrient uptake and translocation at different growth stages and in response to environmental stresses.

Secondly, from one scan multiple leaf properties can be simultaneously modeled and estimated (given the models for the target properties are already built). This multi-sensing capability of VIS–NIR–SWIR is desirable for high throughput phenotyping. It further improves measurement speed and reduces cost (where multiple traits can be obtained from a single scan). Field-deployable VIS–NIR–SWIR instruments are commercially available. These commercial instruments are equipped with suitable accessories (such as a leaf clip) to facilitate the collection of high-quality hyperspectral data in the field. This eliminates the need to collect, handle and preserve physical leaf samples (during which the plant leaf could change its properties), as well as conducting subsampling for lab analysis of different traits, and potentially leads to more accurate measurements.

### Building VIS–NIR–SWIR spectral libraries to support high-throughput plant phenotyping research

It is important to note that VIS–NIR–SWIR does not measure leaf physiological or chemical properties directly. Rather, it is a data-driven approach where multivariate models (such as PLSR and SVR models) are developed to make the estimation. Model calibration, which requires a set of samples to be measured with the reference methods, is the most expensive part of VIS–NIR–SWIR. In research settings, researchers often measure more than 50% of their samples for model calibration. However, for the VIS–NIR–SWIR technique to be adopted at the commercial scale, it is neither practical nor economical to develop a calibration set for each individual project. Here we propose the development of plant leaf VIS–NIR–SWIR spectral libraries in the plant phenotyping community. These libraries will include both hyperspectral data and lab data, as well as pre-trained spectroscopic models. In this fashion, individual scientists or breeders do not need to develop a model calibration set. Rather, they can use the spectral libraries (and pre-trained models) to make predictions for their samples, therefore improve the cost-effectiveness and throughput of their analyses.

Developing such plant leaf VIS–NIR–SWIR spectral libraries is not trivial and requires thorough planning and long-term collaboration from multiple research labs and entities. Some major factors to be considered are the plant species (maize, wheat, sorghum, etc.), leaf properties to be predicted (chlorophyll content, N, photosynthetic parameters, etc.), and modeling approaches employed. A model optimized for predicting leaf N content of maize plants might not work well for wheat or sorghum. Even within maize, models generated from one panel of plants under certain experimental condition may not work well for another experimental condition. Maintaining good records of metadata and practicing QA/QC for each dataset to be included into the VIS–NIR–SWIR spectral library are critical, such that users can evaluate the quality and applicability of the models. The library should be searchable, so that users can search for the most appropriate samples in the library to form the calibration set of their own and train the models on the fly.

Imagine that a plant breeder is carrying a portable VIS–NIR–SWIR instrument to measure hyperspectral leaf reflectance of plants in the field. The instrument was connected to a spectral library which enables the breeder to make real-time estimation of an array of leaf properties. The researcher will be able to measure hundreds of plots quickly, with virtually no additional cost or effort. When returning to office, he/she will readily use these real-time predicted leaf properties for more sophisticated analysis such as heritability analysis, QTL mapping or genomic prediction. We believe that if such a VIS–NIR–SWIR spectral library is developed, it will contribute to high throughput plant phenotyping and accelerate the targeted crop improvement (particularly for leaf physiological and chemical traits) in very substantial ways.

## Conclusion

In this study, we investigated the usefulness of VIS–NIR–SWIR leaf reflectance to estimate six leaf physiological and biochemical properties of maize plants. We showed that leaf chlorophyll content and nitrogen content were estimated accurately. Leaf water content, specific leaf area, and potassium content were estimated with moderate accuracy; and phosphorus was estimated with low accuracy. We also showed that Partial Least Squares Regression and Support Vector Regression gave higher prediction accuracy than Vegetation Indices. It is concluded that VIS–NIR–SWIR leaf reflectance can be a powerful tool for low-cost, nondestructive and high-throughput analysis of leaf physiological and biochemical traits. Development of a VIS–NIR–SWIR leaf spectral library would great benefit the plant phenotyping community for the research of leaf traits.

## Data Availability

The datasets generated and analyzed during the current study are available from the corresponding author on reasonable request.
